# Body weight-dependent foot loads, assessed in terms of BMI and adiposity, in school-aged children: a cross sectional study

**DOI:** 10.1038/s41598-020-69420-1

**Published:** 2020-07-23

**Authors:** Beata Szczepanowska-Wolowiec, Paulina Sztandera, Ireneusz Kotela, Marek Zak

**Affiliations:** 1 Institute of Health Sciences, Collegium Medicum, The Jan Kochanowski University, 25-317 Kielce, Poland; 2 Rehabilitation Clinic, Provincial General Hospital, 25-310 Kielce, Poland; 3 Institute of Medical Sciences, Collegium Medicum, The Jan Kochanowski University, 25-317 Kielce, Poland; 4 Central Clinical Hospital of the MSWiA, 02-507 Warsaw, Poland

**Keywords:** Health care, Paediatrics, Paediatric research

## Abstract

Whereas inherently vulnerable structure of both a child's and an adolescent's foot, characteristic for its dynamic, developmental stage, is particularly exposed to numerous environmental factors, excessive body weight gain may potentially become a crucial causal factor, bringing on a cascade of adverse effects throughout the body, e.g. disorders of the skeletal-articular system, gait alterations, abnormally excessive loading of the plantar zones of the foot, and consequently serious postural defects, especially in later life. Since obesity, aptly dubbed the scourge of the 21st c., directly impacts the way the foot biomechanics are developed, whereupon the actual paradigm of foot loading becomes subject to numerous, adverse modifications, the present study focused on gaining an in-depth insight into prevalent association of BMI, adipose tissue content in body composition, and the actual distribution of foot loads in the school-aged children. Since body weight, the simplest anthropometric indicator, is actually non-indicative of the proportion of adipose tissue within body composition, a number of modern, non-invasive diagnostic methods were applied by the investigators to have this deficit effectively addressed, inclusive of comprehensively mapping out the actual load distribution in the plantar zones of the foot.

## Introduction

The World Health Organization (WHO) acknowledges obesity to be the most common metabolic disease, aptly dubbing it a global scourge of the 21st c. This metabolic phenomenon has also affected children and teenagers. The number of overweight and obese cases in the youngsters aged 5–19 years increased from 4% in 1975 to over 18% in 2016. Obesity is a natural consequence of an imbalance between energy supply and its expenditure. Excessive dietary intake, usually combined with low physical activity, appreciably contribute to an increased weight gain through boosting the proportion of adipose tissue within the body composition^1–3^.

Excessive body weight appreciably contributes to abnormal motor development, agility, and overall coordination of movements, as well as adversely affects the development of the skeletal-articular system, which may consequently result in postural defects. Last but not least, excessive body weight may also be a crucial causal factor in various abnormalities encountered in the feet, i.e. abnormal foot loads^4–6^.

There are many studies corroborating detrimental effect of increased body weight on foot loads and attendant deformities^4–6^. Numerous reports imply that overweight and obesity may well be instrumental in developing flat-footedness^7,8^, even though there are also studies which call this particular assertion into question^9^.

Cousins et al.^10^, while examining overweight and obese children observed that these children put more load on the metatarsal, in particular onto 2–5 metatarsal bones, than their peers with normal body weight. On the other hand, Buldt et al.^11^ having conducted studies of adults with normal, flat, and cavus foot (i.e. a foot of high medial longitudinal arch), observed that flat-footed individuals (i.e. with abnormally low longitudinal arch) put more load on the lateral part of the forefoot than the ones with normally structured feet. Butterworth et al.^4^ examined adults and reported an association between an increased body weight and an increased load on the forefoot, and its metatarsal part. Discrepant results prompted us to undertake the present study, with a view to gaining some more conclusive insights into this particular issue.

The foot, meant as a support for the locomotor system, should be fully compliant with its inherent ergonomics. In an upright stance, load distribution in a normally vaulted foot is as follows, i.e. the heel takes over 60% of the body weight, the metatarsal part—8%, and the forefoot—32%^12^. Even slight alterations in the foot biomechanics may affect this distribution structure. Regular monitoring of feet loading is therefore believed to appreciably gain in diagnostic significance, as well as in terms of effective prevention. A stabilometric platform was used as a research tool of choice for assessing the distribution of foot loads in the plantar part of the foot^13^.

Application of modern, non-invasive diagnostic methods facilitates an in-depth assessment of body weight, inclusive of determining the actual proportion of adipose tissue within the body composition. It may also effectively aid the assessment of pressure (load) distribution on the plantar part of the foot, which has prompted us to focus our research around this particular issue^14^.

The study aimed to gain an insight into prevalent association of BMI, adipose tissue content in body composition, and distribution of foot loads in school-age children.

## Results

The study group involved 194 children (52.1% girls, 47.9% boys), aged 12–14 years, recruited from both urban and rural environments. The average age was 12.84, average body weight—49.92 kg, and average height—157.6 cm (Table 1).

**Table 1:** Basic characteristics of the study group.

Variable	Girls (n = 101; 52.1%) Mean ± SD	Min–max	Boys (n = 93; 47.9%) Mean ± SD	Min–max	*p*
Body weight in kg	48.8 ± 10.3	26–74	51.2 ± 13.9	29–97	0.56
Body height in m	1.6 ± 0.1	1.3–1.7	1.6 ± 0.1	1.4–1.8	0.24
BMI	19.7 ± 3.2	12.9–28.8	20.2 ± 4.2	13.8–36.5	0.78
Adipose tissue content in %	24% ± 4%	15–35%	20% ± 7%	11–47%	< 0.01

*Mean* arithmetic mean, *SD* standard deviation, *BMI* body weight index, *Min* minimum value, *Max* maximum value, *p* significance level.

The Clarke's angle values comprised in Table 2, facilitating assessment of the longitudinal vaulting of the foot, indicate that in the study group with a lowered foot vaulting this applied to the left foot in 11.3% of the subjects, and to the right foot—in 15.5% of them. There were 7.7% subjects with both left and right hollow foot (cavus foot), whereas no feet with the values below 30° of the Clarke's angle (flat foot) were encountered among them.

**Table 2:** Assessment of the foot's longitudinal vaulting in line with the Clarke’s angle.

Clarke’s angle	Left foot		Right foot	
	n	%	n	%
Lower foot arch	22	11.3	30	15.5
Normal foot	157	80.9	149	76.8
High-arched foot	15	7.7	15	7.7

*n* number of subjects.

Table 3 comprises the assessment of the transverse vaulting of the foot, in due consideration of the Wejsflog index. No statistically significant dependence between the foot vaulting and its loading was established.

**Table 3:** Assessment of the foot's transverse vaulting in line with the Wejsflog index.

Wejsflog index	Left foot		Right foot	
	n	%	n	%
Transverse flat foot	12	6.2	15	7.8
Normal foot	182	93.8	179	92.2

**Figure 1:**
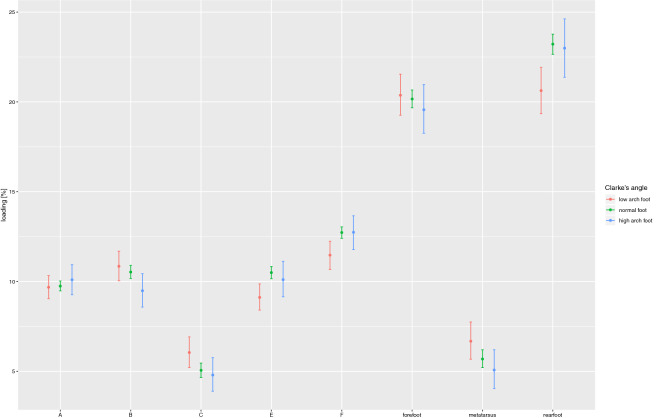
The dependence between foot loading under the static conditions and the Clarke’s angle.

Figure 1 depicts the dependence between the foot loading and a specific foot type. The study subjects with a lowered foot vaulting tend to load the hindfoot much less, as compared to those with a normally vaulted foot. A statistically significant dependence (*p* = 0.001) was established in a single case only. This difference is clearly manifest in both lateral (*p* = 0.005) and medial (*p* = 0.013) type of foot loading. No statistically significant dependence was established for a hollow foot (cavus foot), though.

Significant differences are clearly visible during the foot loading phase:

- Hindfoot—individuals with a lowered longitudinal foot vaulting tend to load this part of the foot significantly less (20.63%), as compared to the ones with normally structured feet (23.21%; *p* = 0.001)
- Zone E—individuals with a lowered longitudinal foot vaulting tend to load this part of the foot significantly less (9.12%), as compared to the ones with normally structured feet (10.5%; *p* = 0.005)
- Zone F—individuals with a lowered longitudinal foot vaulting tend to load this part of the foot significantly less (11.47%), as compared to the ones with normally structured feet (12.73%; *p* = 0.013)

Table 4 shows the average values of the loaded parts of the foot. In the case of zone D, there was a very high sample rate (39% for the left foot, 65% for the right foot), even though this area was not loaded at all.

**Table 4:** Loading of respective zones of the foot within the entire study group.

Foot zones	Left foot Mean ± SD	Right foot Mean ± SD
A	9.5 ± 2.4	10.2 ± 2.0
B	10.5 ± 3.2	10.9 ± 2.5
C	5.3 ± 2.28	5.6 ± 3.0
D	1.0 ± 1.2	0.5 ± 0.9
E	11.2 ± 2.8	9.8 ± 2.5
F	12.4 ± 2.9	12.4 ± 2.6

A—lateral forefoot, B—medium forefoot, C—lateral midfoot, D—medium midfoot, E—lateral hindfoot, F—medium hindfoot.

Linear mixed models were applied, with a view to determining how gender, BMI, and adipose tissue content determined the distribution of foot loads forces under the static conditions.

All foot loading level dependent variables were analysed in terms of normal distribution. For most variables a Box–Cox transformation was applied. Only with regard to the percentage of hindfoot loading this transformation was applied, as the original variable values were used instead. In the case of zone D, the model was not adjusted. Both the left and right foot loads were taken into account in the respective models. Consequently, the observations were not independent, a study subject participant was added as a random effect.

**Figure 2:**
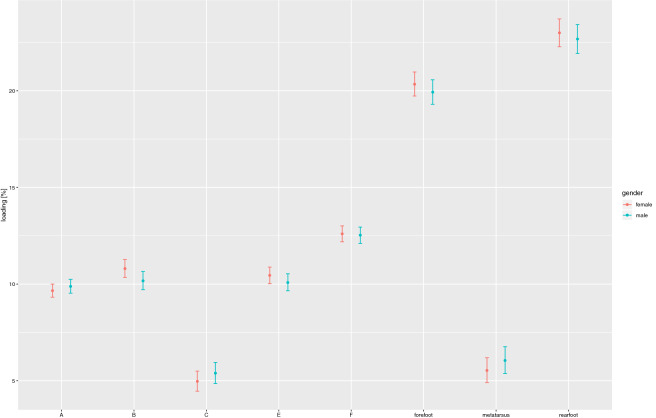
The dependence between foot loading under the static conditions and the subjects' gender.

**Figure 3:**
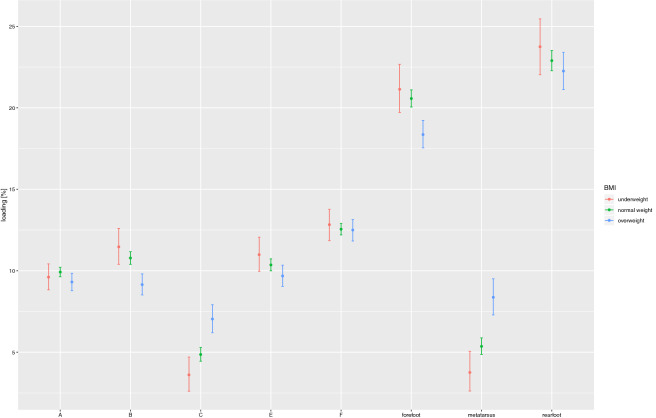
The dependence between foot loading under the static conditions and BMI.

In view of the assessment of several foot zones, the level of significance was adjusted with the aid of Šidák correction for multiple comparisons. The results for the Box–Cox transform variables were re-transformed using the reverse transformation, so as to secure the final results. A comparison of the foot loads in different batches in terms of gender yielded no significant differences. Girls and boys aged 12–14 years were found to load their feet in much the similar way. Figure 3 and Table 3 show the mean values of the foot loads in different zones with 95% confidence intervals, as stratified by gender (Fig. 2, Table 5).

**Table 5:** Breakdown of foot loading into five foot zones under the static conditions and the subjects' gender.

Foot zone	Gender	Mean (%)	CI 95%	*p*
A	K	9.66	(9.32%, 10.00%)	0.908
	M	9.88	(9.53%, 10.24%)	
B	K	10.80	(10.34%, 11.27%)	0.278
	M	10.17	(9.71%, 10.65%)	
C	K	4.97	(4.46%, 5.50%)	0.797
	M	5.39	(4.85%, 5.95%)	
E	K	10.45	(10.02%, 10.88%)	0.745
	M	10.08	(9.65%, 10.53%)	
F	K	12.60	(12.19%, 13.01%)	1.000
	M	12.53	(12.10%, 12.95%)	

CI 95%—confidence interval

Due to the small size of the study group in the obesity category, based on BMI, for the analysis making use of the linear models, the mixed *overweight* and *obesity* categories were combined (Table 6).

**Table 6:** Breakdown of BMI into constituent categories.

BMI breakdown	n	%
Underweight	18	9.3
Reference value	136	70.1
Overweight + obesity	40 (34 + 6)	20.6

*n* number of subjects.

Figure 3 shows the dependence between foot loading and BMI. Statistically significant differences are visible with regard to foot loading.

- forefoot—overweight subjects *significantly less* (18.36%) loaded that part of the foot, as compared to those whose BMI was both standard (20.57%; *p *< 0.001), or indicated underweight (21.14%; *p *= 0.003)
- metatarsal—overweight subjects (8.37%) significantly more loaded that part of the foot, as compared to underweight ones (3.76%; *p *< 0.001), and those remaining well within the standard value range (5.36%; *p *< 0.001)
- Zone B—overweight subjects (9.15 %) significantly less loaded that part of the foot, as compared to those who remaining well within the standard value range (10.78 %; *p *< 0.001), and indicating underweight (11.47 %; *p *= 0.002)
- Zone C—overweight subjects (7.04%) significantly more loaded that part of the foot, as compared to the underweight ones (3.61%; *p *< 0.001), and those remaining well within the standard value range (4.86%; *p *< 0.001)

The assessment of the dependence between BMI and foot loading indicates that overweight subjects, as compared to those with normal body weight and the underweight ones, tend to load more the metatarsal part of the foot, i.e. zone C (*p* < 0.001).

In terms of adipose tissue content analysis, we excluded 4 cases which fell into the *underweight* category (pursuant to breakdown into constituent categories), as well as had the *overweight* and *obesity* categories combined into a single one (Table 7).

**Table 7:** Breakdown of adipose tissue into constituent categories.

Adipose tissue	n	%
Reference value	142	73.2
Overweight (overweight + obesity)	48 (30 + 18)	24.7

**Figure 4:**
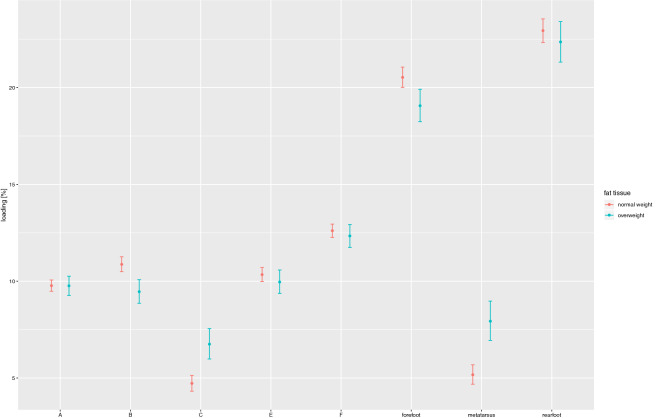
The dependence of foot loading under the static conditions on adipose tissue content.

Figure 4 shows the dependence between foot loading and a proportion of adipose tissue in body weight. Much as in the case of BMI, statistically significant differences are visible with regard to foot loading.

- forefoot—overweight subjects loaded the forefoot on an average level (19.06%), whereas the ones remaining well within the standard value range loaded this part at the 20.53% level; the difference between respective groups being statistically significant (*p* = 0.013)
- metatarsal—overweight subjects loaded the metatarsal significantly more (7.93%), as compared to the ones remaining well within the standard value range (5.17%; *p* < 0.001)
- Zone B—overweight subjects loaded Zone B significantly less (9.46%) than the ones remaining well within the standard value range (10.87%; *p* = 0.001)
- Zone C—overweight subjects loaded Zone C significantly more (6.75%) as compared to the ones remaining well within the standard value range (4.72%; *p* < 0.001)

The assessment of dependence between adipose tissue content and foot loading also indicates that the subjects with a higher adipose tissue content, as compared to those remaining well within the reference range, tend to load more the metatarsal part of the foot, i.e. zone C (*p* < 0.001).

Statistically significant dependence was established between BMI, adipose tissue content and foot loading for the lateral metatarsal part of the foot (zone C).

By way of summing up, it should also be highlighted at this juncture that no significant dependence whatsoever was established between the actual type of foot vaulting and the foot loading paradigm.

## Discussion

In order to have their locomotor and supporting function implemented effectively, the lower limbs must have a correctly developed anatomical structure, especially the feet. While appraising oneself of the biomechanics of a human body, foot pressure and foot load as the key factors should primarily be taken into account. Specifically targeted studies, conducted both in adults and children, highlight the fact that abnormalities in the feet consequently affect the skeletal-articular system at large^15–18^. Presently, wide availability of modern, non-invasive test platforms facilitates measuring the pressure of the plantar part of the foot, while standing and walking. Effective measurement of the actual foot load distribution is aided by the division of the plantar part of the foot into specific load zones. The basic division separates the foot into the forefoot and the hindfoot, whereas a more detailed one, depending on respective investigators, allows for 12 specific zones^19^.

According to WHO, obesity is an increase in body weight through pathological growth of adipose tissue, far exceeding the physiological needs and adaptability of the body. Body weight is comprised of the weight of muscles, bones, extracellular water, and adipose tissue. The simplest anthropometric indicator, i.e. body weight, makes it unfeasible to determine the actual proportion of adipose tissue in body composition^20^.

In clinical practice, assessment of obesity is based on the BMI (Body Mass Index), which is calculated by dividing individual body weight in kg by the height in metres squared. Another indicator is the WHR (waist/hip ratio), a measurement of central obesity. The studies focused on accurate measurement of body fat content, make use of various methods, e.g. electrical bio-impedance, dual-energy X-ray absorptiometry (DXA), computed tomography with planimetric evaluation, nuclear magnetic resonance imaging, ultrasound methods, or isotope-based testing^20^.

The present study deals with the effect of obesity and overweight on foot loading. The Kułaga classification^21^ was applied to assess the division of body weight and nutritional status in relation to BMI. In the present study, BMI centile distribution was used, as developed by Kułaga et al.^21^, pursued under the OLAF and OLA research constraints. The present research project involved 22,211 children from Poland. When making use of the centile distribution approach, it seems only prudent to refer to the studies of a specific population which take due note of the impact of environmental factors characteristic of a particular ethnicity^22–24^.

A simple and quick way of assessing BMI may inherently be burdened with a number of inaccuracies, though. Whilst striving to preclude any inadequate assessment, due references to the body fat content, division into underweight, norm, overweight, and obesity, were also taken into account in the present study; individual body fat content having been assessed in line with the classification adopted by McCarthy et al.^25^.

Obesity, popularly dubbed the epidemic of our times, directly impacts the way the foot's biomechanics are developed. The foot, an essential component of the locomotor system, is shaped in a way totally unique for every individual. The delicate structure of a child's foot is quite susceptible to a variety of adverse, environmental factors. Any excessive body weight gain consequently results in a cascade of adverse effects within the entire body, primarily manifest in the locomotor system^26^.

Many investigators believe that being prone to obesity is the factor actually determining functional capacity of the feet. Brzezinski et al.^26^, whilst examining 6,992 children aged 8–12 years, reported the lower limb defects in 90.2% of obese children, i.e. 19.5% of the entire study group. They also established that likelihood of lower limb defects increases with weight gain. Woźniacka et al.^27^ in their study of 1,115 children also noted a significant correlation between the foot arching and obesity.

Making use of the Clarke’s angle in assessing the longitudinal vaulting of the feet, the Authors observed a higher incidence of high-arched feet, especially among the underweight children. High-arched foot was encountered in 66.5% of the study subjects (right foot), and in 61.4% (left foot), respectively. Flat-footedness was observed the least frequently in the group under study, although it was encountered far more frequently in the overweight and obese children, most often in the boys. No similar associations were reported in the study group, as the high-arched foot was observed in 7.7% of children (left foot), and in 7.7% (right foot), respectively. A smaller number of diagnosed abnormalities within the feet may well be attributable to a smaller size of the study sample. On the other hand, overweight/obesity was far more often diagnosed in our own research, especially in boys, much like in the study by Woźniacka^27^.

In our own study, it was established that girls had a higher adipose tissue content than boys, which proved statistically significant, as well as fully consistent with the findings of Bredella^28^.

Lui et al.^29^ did not report any differences between gender and foot loads in the group of healthy individuals (age range 6–16 years). Similar conclusions were drawn by Phethean and Nester^30^ in their study of 98 healthy children, aged 4–7 years. Also Gijon-Nogueron et al.^31^, following their examination of 1,798 children, aged 6–12 years, did not find any association between gender and age and static foot posture. The effect of age and gender on the load in the plantar part of the foot was noted by Demirbüken et al.^32^, who studied 524 individuals, aged 11–14 years. They concluded that gender- and age-dependent changes in the foot loads may actually be a potential risk factor for foot abnormalities. In our own study, no correlation whatsoever was established between gender and the foot load. This might well be due to a less numerous study sample, so further research into the issue should obviously be pursued.

Cousins et al.^10^, when comparing 22 overweight children, 22 obese children, and 56 children of normal body weight, highlighted the incidence of higher loads during walking in overweight and obese children. They reported the differences in foot loading in overweight children, i.e. these children were reported to put appreciably more load on 2–5 metatarsal bones; a similar trend having been observed in obese children.

In our study, there is a significant statistical difference in the loading of the metatarsal part of the foot in the overweight children, as compared to the underweight individuals and those of normal body weight. The subjects put more load on this area, especially on Zone C (lateral part of the metatarsal). Mickle et al.^33^, while comparing gait in overweight/obese pre-school children, observed higher loading in the metatarsal area in the overweight/obese children. This phenomenon also attracted our attention, even though in our study the subjects were of a different age.

Park et al.^3^, while examining the foot arching, pressure on the plantar part of the foot, and balance among 52 young adults, found more abnormalities in the obese adults than in those characterised by normal body weight. Obese and overweight patients had increased loading on the plantar part of the foot, different thickness of their plantar fascia, and balance problems, as opposed to the ones of normal body weight.

Having reviewed the results of our own studies focused on comparing BMI and foot pressure under static conditions, we noted that overweight and obese individuals put more load on the metatarsal, and part C, than children with normal body weight or underweight (significant correlation *p* < 0.001). Assessment of the relation between the foot load and adipose tissue content in overweight and obese children also evidenced the increased foot loading on the metatarsal, especially in the C-zone—the lateral side of the metatarsal, to be a significant correlation (*p* < 0.001). There are numerous studies corroborating the assertion that there is no difference between the thickness of adipose tissue pad located in the plantar part of the foot in the overweight/obese children and those of normal body weight^34^. To the best of our knowledge, there are very few reports addressing the relationship between the proportion of adipose tissue in the body composition and the foot structure and its loading paradigm. In our study, we observed that children with adiposity tend to put more load on the metatarsal area of the foot, thus facilitating greater contact between the metatarsal and the ground owing to the lowered longitudinal arching of the foot.

Evans and Karimi^9^, however, having examined 728 children, did not encounter any correlation whatsoever between increased body weight and flat-footedness in children.

Woźniacka et al.^35^, having examined 925 children, reported that the most commonly encountered deformity was a high-arched foot, i.e. in 523 children (56.5%) in the left foot, and in 592 children (64%) in the right foot. Flat-footedness was reported in overweight and obese children. Such a high incidence of high-arched feet was not encountered in our own study, though, which might well be attributable to the fact that our study group was mostly characterized by normal foot vaulting. Hence we searched for the association between increased body weight and foot loading. The results of our own research provide ample evidence that alterations in individual body weight translate into statistically significant effects on the actual loading of respective zones in the plantar area of the foot.

The effect of excessive body weight is appreciably instrumental in developing various ailments within the musculoskeletal system, alterations in gait, the foot structure itself, as well as the altered load on the plantar part of the foot^36–39^. As evidenced throughout our own study, there was a fundamental difference in the biomechanics of foot loading. The impact of overweight and obesity on the abnormal distribution of foot loading was clearly manifest. Pursuit of early diagnostic assessment, and subsequent, specifically targeted intervention, is therefore strongly recommended, with a view to minimising the incidence of attendant musculoskeletal complications.

Such a small percentage of foot deformities in our study subjects gives us grounds to believe that flat-footedness is not such a popular defect after all. One may even venture to say that even a slight change in body weight is immediately reflected in the altered loading of the plantar part of the foot. The most crucial observations supported by this study consist in the fact that in a study group almost homogeneous in terms of longitudinal vaulting of the foot, any alterations in foot loading are owed to higher BMI values and adiposity. This notwithstanding, further in-depth research is obviously required to lend even more credence to the results at issue.

The present study was also subject to certain limitations. The actual testing was pursued in a static position, so its results may on no account be automatically extrapolated onto the dynamic conditions. Even though a small size of the study sample may offer some valid pointers in terms of general direction of the presently pursued investigation, further studies conducted on the sizable populations are required to have our working hypotheses effectively verified.

## Conclusions

No significant correlation was established between the foot load and gender, while the assessment of BMI and foot load dependence in the overweight and obese individuals, as compared to the ones characterised by normal body weight, clearly demonstrated that the former tended to put far more load on the metatarsal; this proving to be significantly correlated. Furthermore, juxtaposition of adipose tissue content within the body weight against the foot load distribution yielded yet another significant correlation, as overweight individuals were found to put far more load on the metatarsal, especially in the C zone of the foot.

## Research design and methods

The survey involved 194 children (101 girls and 93 boys) aged 12–14 years, randomly selected from primary schools, representing both the urban and rural environments of a single province, whose characteristics are presented in Table 1. All study subjects had been granted permission to attend by their parents/guardians.

The following inclusion criteria were adopted: informed consent to participate in the study protocol, full documentation of the study, no disorders in the locomotor system, as assessed through an interview. The exclusion criteria were as follows: no informed consent to participate in the study protocol, incomplete documentation of the study, disorders in the locomotor system, as assessed through an interview, metabolic disorders that might potentially affect the skeletal system^40^.

### Anthropometric measurements

The subjects' body height was measured with the aid of SECA stadiometer (manufactured in Germany 93/42/EWG, 2007/47/WE; measurement accuracy 0.01 m), body mass was measured using Tanita scales (BC-418MA type, manufactured in Japan 93/42/EEC Annex II; measurement accuracy ± 0.1 kg), while the foot load test, carried out in static conditions, was assessed by FreeMed platform, operated by FreeStep Pro software (FreeMed, Sensor Medica, Italy).

The Tanita scales analyses the body mass composition with the aid of electrical bio-impedance technology. The device is fitted with 8 sensors, placed underneath the platform on which a person stands barefooted, as well as in the handles to be held during the procedure. The measurement of electrical bio-impedance, dependent upon respective electrical conductivity of specific body tissues, facilitates the actual assessment of whether a person happens to be undernourished, obese, or just overweight. The BMI index was divided making use of the percentile nets developed by Kułaga et al.^22^ for Polish children, as well as for evaluation of adipose tissue content^25^.

Making use of a 2D podoscan, the loading of a plantar part of the foot was assessed under static conditions. A computer programme analyses the actual image of the foot, determining its length, width, angles, and axes. A study subject stands on the platform barefooted, feet set parallel, lower limbs straight, upper limbs hanging loose along the torso^40^. The Clarke’s angle and Wejsflog index were assessed. The Clarke's angle below 30º is construed synonymous with flat-footedness, values within the 31°–41° range denote a foot with a lowered longitudinal vaulting, the ones within the 42°–54° range attest to its anatomically correct vaulting, whereas the angle over 55° attests to a high-arched foot^17,18,40^. Taking into account the values of the Wejsflog index, the transverse vaulting of the foot is assessed. The ratio of the foot's length to its width should be 3:1. Values of e.g. 2.10 attest to a transverse flatness of the foot, whereas the 2.97 ratio confirms an anatomically correct transverse vaulting of the foot^17,18,40^.

### Distribution of foot loads

The following stage of the testing was carried out with the aid of the FreeMed Maxi stabilometric platform, which facilitates effective assessment of distribution of a foot load. It may also be applied to test individual balance, as well as identify any deficits in visual-motor coordination. Functional assessment of the foot may be carried out both under static and dynamic conditions. During the test, the subjects stood on the device barefoot, at ease, with the upper limbs hanging freely along the torso, feet parallel, the eyes fixed on the mark right in front.

A sample of the study charts is comprised in Fig. 4

**Figure 5:**
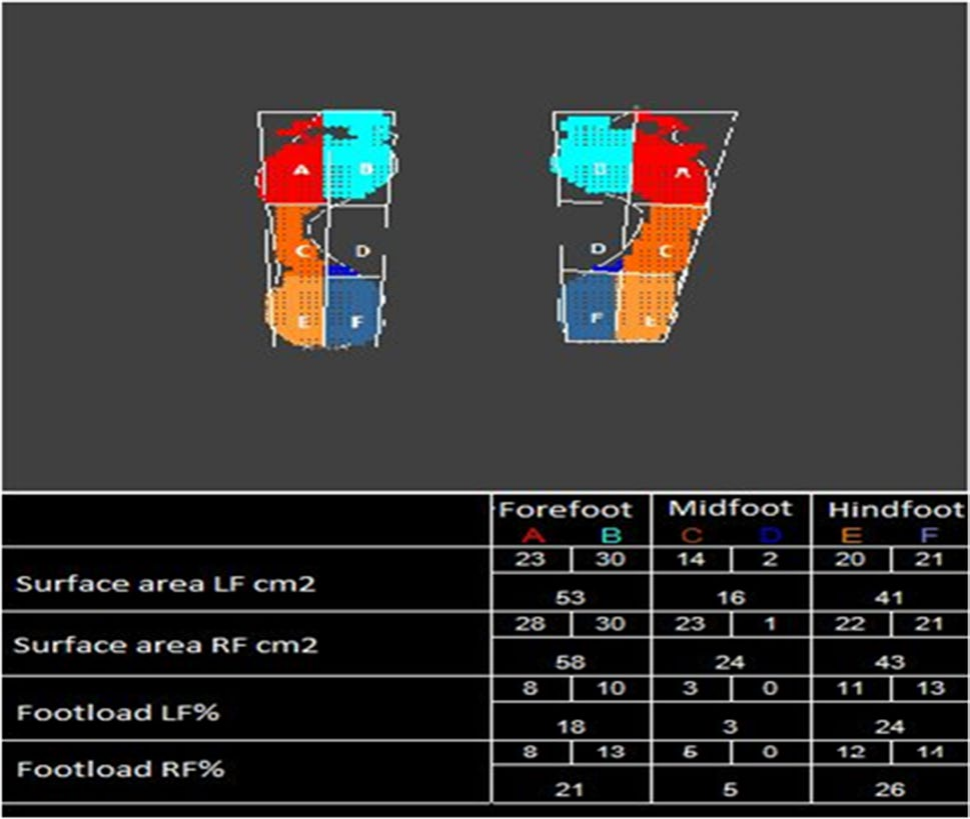
An example of the result of a foot load test under static conditions; demarcation of respective zones within the plantar part of the foot. *Source*: Own research material.

When testing under the static conditions, the foot was divided into six zones, i.e. forefoot—zones A and B, metatarsal—zones C and D, hindfoot—zones E and F (Fig. 5).

The following zones on the plantar part of the foot were assessed:

- A—lateral forefoot
- B—medium forefoot,
- C—lateral midfoot
- D—medium midfoot
- E—lateral hindfoot
- F—medium hindfoot (Fig. 5).

### Statistical analysis

Statistical analyses were carried out using R version 3.5.0 software package and MS Office Excel. With regard to all variables under study, basic metrics of descriptive statistics were used i.e. arithmetic mean, standard deviation, minimum and maximum for the entire group. The comparison of characteristics between the groups was preceded by the Kolmogorov–Smirnov test, with a view to assessing normality of the distribution. When the distribution was normal, the Welch test was applied, whereas otherwise the non-parametric Mann–Whitney-Wilcoxon test was used. Impact of such variables as gender, BMI, and adipose tissue content on the foot load distribution was assessed through the linear mixed models. The assessment of foot loading was analysed in terms of normal distribution. For most variables the Box–Cox transformation was applied. The assessment of several foot zones required the application of Šidák correction for multiple comparisons.

## The compliance statement

All research methods comprised in the study protocol were pursued/implemented in full compliance with pertinent guidelines, regulations, and applicable legislation in place.

## Ethics approval

The study design and protocol was granted approval and duly endorsed by the Bioethics Review Committee, established in pursuance of pertinent statutory constraints at the Faculty of Medicine and Health Sciences, The Jan Kochanowski University in Kielce, Poland, following rigorous appraisal of the investigators' application for ethics approval, carried out on June 20, 2016 (Ethics Approval Ref. No. 26/2016).

## Informed consent

Written informed consent was obtained from the parents/guardians of the minor study subjects for their attendance in the study protocol. It was based on the detailed information on the actual aims and research methods to be used, having prior been furnished to them by the authors.
